# HEALTH TECHNOLOGY ASSESSMENT OF MEDICAL DEVICES: A SURVEY OF NON-EUROPEAN UNION AGENCIES

**DOI:** 10.1017/S0266462315000185

**Published:** 2015

**Authors:** Oriana Ciani, Britni Wilcher, Carl Rudolf Blankart, Maximilian Hatz, Valentina Prevolnik Rupel, Renata Slabe Erker, Yauheniya Varabyova, Rod S. Taylor

**Affiliations:** Evidence Synthesis & Modelling for Health Improvement, Institute of Health Research, University of Exeter Medical School; Centre for Research on Health and Social Care Management (CERGAS), Bocconi Universityo.ciani@exeter.ac.uk; Institute of Health Research, University of Exeter Medical School; Hamburg Center for Health Economics, University of Hamburg; Institute for Economic Research (IER); Hamburg Center for Health Economics, University of Hamburg; Evidence Synthesis & Modelling for Health Improvement, Institute of Health Research, University of Exeter Medical School

**Keywords:** Medical devices, Health technology assessment, Reimbursement

## Abstract

**Objectives:** The aim of this study was to review and compare current health technology assessment (HTA) activities for medical devices across non-European Union HTA agencies.

**Methods:** HTA activities for medical devices were evaluated from three perspectives: organizational structure, processes, and methods. Agencies were primarily selected upon membership of existing HTA networks. The data collection was performed in two stages: stage 1–agency Web-site assessment using a standardized questionnaire, followed by review and validation of the collected data by a representative of the agency; and stage 2–semi-structured telephone interviews with key informants of a sub-sample of agencies.

**Results:** In total, thirty-six HTA agencies across twenty non-EU countries assessing medical devices were included. Twenty-seven of thirty-six (75 percent) agencies were judged at stage 1 to have adopted HTA-specific approaches for medical devices (MD-specific agencies) that were largely organizational or procedural. There appeared to be few differences in the organization, process and methods between MD-specific and non–MD-specific agencies. Although the majority (69 percent) of both categories of agency had specific methods guidance or policy for evidence submission, only one MD-specific agency had developed methodological guidelines specific to medical devices. In stage 2, many MD-specific agencies cited insufficient resources (budget, skilled employees), lack of coordination (between regulator and reimbursement bodies), and the inability to generalize findings from evidence synthesis to be key challenges in the HTA of medical devices.

**Conclusions:** The lack of evidence for differentiation in scientific methods for HTA of devices raises the question of whether HTA needs to develop new methods for medical devices but rather adapt existing methodological approaches. In contrast, organizational and/or procedural adaptation of existing HTA agency frameworks to accommodate medical devices appear relatively commonplace.

Health technology assessment (HTA) seeks to provide policy makers with information on the clinical and economic value of health technologies (including pharmaceuticals, medical devices (MDs), clinical procedures, and organizational systems used in health care) to inform their reimbursement or coverage decisions (1).

Several surveys have been undertaken to date that describe and compare the HTA activities across organizations and countries (2–6). For example, in 2008, the European Network for Health Technology Assessment (EUnetHTA) published an international survey of HTA organizations examining the barriers and solutions for establishment of the HTA units, their characteristics and processes (6). These previous surveys have tended to focus on drugs rather than non-drug technologies (7), often because “the use of HTA information in the reimbursement of pharmaceuticals is currently the most advanced area of research in the discipline” (3). However, the report by Wilsdon and Serota showed that several countries have HTA systems in place with a mandate to include non-drug technologies, including MDs (8). This report also confirmed that the number of HTA reports devoted to non-drug technologies remain in the minority.

The evaluation of non-drug technologies, in particular MDs, may pose different challenges to those of pharmaceuticals. Compared with drugs, undertaking randomized controlled trials are often more difficult, product modification occurs more frequently, pricing is more dynamic, and clinical outcomes are more depending on training, competence, and experience (“learning curve”) of the operator (9;10). It is often difficult to disentangle procurement costs (including associated infrastructure) and running costs (including maintenance and consumables) of MDs (11).

The aim of this study was to undertake a survey of non-European Union (EU) HTA agencies to characterize and contrast their activities for MDs in terms of: (i) organizational structure; (ii) processes, that is, standard operating procedures in use; and (iii) methods, that is, scientific methodologies and assessment in place. This study was undertaken as part of the MedTecHTA project (www.medtechta.eu), funded under the EU's 7th Framework Program (FP7). Under the conditions of this EU-FP7 funding, the MedTecHTA project was directed to undertake a survey of MD activities in non-EU HTA agencies. The EU-FP7 funded ADVANCE-HTA project (www.advance-hta.eu) project is responsible for a parallel survey of MD activity in EU HTA agencies.

## METHODS

### Selection of HTA agencies

We considered institutionalized HTA activities (12) and selected HTA organizations based on their membership, as of February 2013, of European network for HTA (EUnetHTA), Health Technology Assessment International (HTAi), International Network of Agencies for Health Technology Assessment (INAHTA) or World Health Organization (WHO) Collaborating Centres for HTA. In addition, we performed a search to identify additional HTA agencies who were not potential members of these networks. To be included, organizations had to be an HTA agency based on the HTA definition proposed by Kristensen (13). Based on this definition, patient and industry organizations were excluded.

### Development of an Evaluative Framework

Our assessment of HTA for MDs was based on the key principles for the improved conduct of HTA proposed by Drummond et al. (14). HTA activities were evaluated from three perspectives: *Organizational structure*: what are the HTA roles of the organization and how is it organized and governed? (e.g., separate department or allocation of resources or deployment of people to the HTA of MDs versus drugs); *Processes*: how does the organization conduct HTA and involve stakeholders? (e.g., separate decision-making committee, degree of stakeholder interaction or amount of time it time of completion of HTA versus drugs); and *Methods*: what methodologies are used for an HTA? (i.e., specific scientific method guidelines for assessing evidence related to MDs versus drugs).

We undertook a two-stage data collection process. In stage 1, we performed: (i) a review of the literature to understand the HTA system in each country, (ii) a review of agency Web sites using a standardized questionnaire, and (iii) review and validation of the completed Web site questionnaires by a senior representative of the agency. In stage 2, we conducted semistructured telephone interviews with key informants of the agencies that were identified at stage 1 as having developed specific HTA activities for MDs (see Supplementary Figure 1).

#### Stage 1: Literature Review, Web Site Data Collection, and Verification of Results

First, we reviewed published literature describing the HTA system in each of the included countries to understand the interactions and players involved from a macro perspective. This involved a Medline (PubMed) search (“country” AND (“Health Technology Assessment” OR “HTA”)). From this information, we developed a “map” summarizing the links between HTA agencies and other key stakeholders (e.g., regulatory agencies) (15;16).

A questionnaire based upon a literature review of HTA of MDs and previous HTA survey (8;17;18) was developed to collate information available of the agencies' Web sites. The questionnaire consisted of fifty items (forty-four closed and six open questions) from four sections (i.e., Section A - agency information, Section B - structure of HTA agency, Section C - agency HTA processes, and Section D - agency HTA methods. A copy of this Web-site assessment questionnaire is available from authors). Data extraction was performed by one researcher and checked by a second.

For verification of the collected data, a key strategic individual within each agency (e.g., Chief Operating Executive, Head of HTA Agency) was e-mailed a copy of our completed Web site questionnaire assessment and the country level HTA map. After verification, if applicable, these individuals were asked if they would participate in a follow up semistructured interview.

#### Stage 2: Semistructured Interviews of Selected HTA Agencies

Interviews were conducted in the subgroup of HTA agencies identified from Stage 1 to have MD-specific processes in terms of their structure, processes, or methods. The objective of the interviews were: (i) to clarify items reported in the Web based data collection process and (ii) to determine how the organizational framework mitigates challenges associated with performing HTA on MDs. If individuals did not respond to the initial invitation e-mail letter and the reminders, other key informants of the agency were identified and asked if they would participate. Interviews were by telephone and semistructured in format, lasting approximately 1 hour, and conducted by two members of the research team. We sought permission to audio-record at the outset of each interview.

### Data Analysis

Data collected from the stage 1 analysis of agency Web sites is presented descriptively. Categorical and continuous data were summarized as frequencies and percentages or as medians and ranges, respectively. Stage 1 results are aggregated across all agencies and presented in a detailed tabular form. Given the objective of this study, aggregate results are also presented separately for “MD specific” and “non–MD-specific” agencies.

Stage 2 audio-recorded interviews collected from MD-specific agencies were transcribed. Answers to open questions were reviewed to identify major topics that were recurrently reported by the interviewees. These topics were then mapped to the three HTA perspectives outlined above, that is, agency structure, processes, and methods. Individual interviews were then searched for certain expressions (e.g., “efficient,” “challenge,” or “suggest”) reflecting either positive or negative opinions/recommendations. Those expressions were then linked to the major themes and coded using NVivo 10 qualitative data analysis software. To summarize the frequency of item occurrence, the following categorization was assigned: “some” (10–30 percent of respondents), “many” (31–65 percent), and “most” (66–100 percent) (19).

## RESULTS

### Selection of Included HTA Agencies

A total of 135 HTA agencies were identified; after applying our exclusion criteria, 36 agencies across 20 non-EU countries were included for analysis (see Supplementary Figure 2).

#### Stage 1: Web Site Data Collection

**Figure 1.**
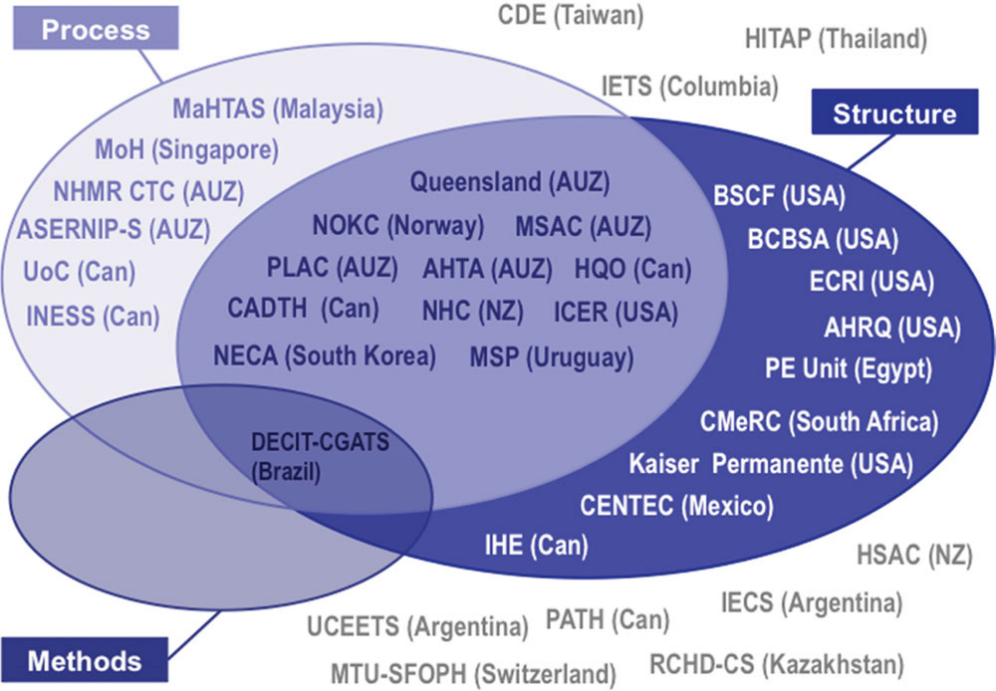
Classification of HTA agencies as non–MD-specific or MD-specific (in terms of process, structure, or methods).

Web site data collection was completed for all thirty-six HTA agencies between June and September 2013; thirty-one (86 percent) data collection sheets were validated by an agency representative. Twenty-seven of the thirty-six agencies were considered to be “MD-specific” because they reported MD-specific information about either their organizational structure (21; 58 percent), processes (18; 50 percent), or methods (1; 3 percent) in stage 1 (Figure 1). The most common model of MD-specific organizational structure was an agency that retained a broad HTA remit (i.e., assessed the full range of health technologies including drugs, diagnostic, public health interventions) but had also developed, within the organization, a specific staff grouping with collective expertise in medical device HTA (e.g., Canadian Agency for Drugs and Technologies in Health (CADTH)). In contrast, the Medicines Advisory Services Committee (MSAC) in Australia is an example of agency established with specific remit for the HTA of devices (along with medical services). Various MD-specific processes were seen across agencies but could be broadly characterized as development of specific HTA process pathways for assessment of devices processes, for example, specific committees for the appraisal of device technologies.

### Structure

The organizational structures of the MD- and non–MD-specific HTA agencies are compared in Table 1. The two groups of agencies appeared broadly similar in terms of their funding, staffing and other element of structure. There were two important differences related to handling of MDs. First, whereas the remit of all HTA agencies included the evaluation of MD technology, a higher proportion of MD reports were produced by MD-specific than non–MD-specific agencies (51 percent versus 10 percent). Second, MD-specific agencies were more likely to apply a classification system when assessing MDs (e.g., US Food and Drug Administration (FDA) class III - life-supporting and life-sustaining products and high-risk devices) than non–MD-specific agencies (56 percent versus 33 percent).

**Table 1.** Summary of Organizational Characteristics of Non-EU Agencies

	All agencies		MD specific		Non-MD specific	
	(*N* = 36)^a^		agencies (*N* = 27)^a^		Agencies (*N* = 9)^a^	
Characteristics	*N*	Median (range)	*N*	Median (range)	*N*	Median (range)
Annual funding (million USD)	20	2.1 (0.01–24.2)	15	2.3 (0.01—24.20)	5	1.0 (0.4–21.0)
Number of staff	31	25 (3–150)	24	19 (3–150)	7	30 (19- 66)
	*N* (%)		*N* (%)		*N* (%)	
Type of organization						
National/Central/Federal Government	16 (44)		13 (48)		3 (33)	
Local/Provincial/State Government	2 (6)		2 (7)		0 (0)	
Academia/university	4 (11)		3 (11)		1 (11)	
Compulsory health care insurance (public)	0 (0)		0 (0)		0 (0)	
Private medical insurance	1 (3)		1 (4)		0 (0)	
Hospital	1 (3)		1 (4)		0 (0)	
Professional association	0 (0)		0 (0)		0 (0)	
Other not-for-profit	7 (19)		4 (15)		3 (33)	
Other	5 (14)		3 (11)		2 (22)	
Source of funding						
National/Local Government^b^	31 (86)		22 (81)		9 (100)	
Private institutions^b^	8 (22)		6 (22)		2 (22)	
Donor agencies (foundations, research funding bodies, international agencies)^b^	7 (19)		3 (11)		4 (44)	
Other^b^	9 (25)		7 (26)		2 (22)	
Professional background of staff						
Clinical specialist/physician	31 [of 32] (97)		24 [of 25] (96)		7 [of 7] (100)	
Economist	26 [of 32] (81)		20 [of 25] (80)		6 [of 7] (86)	
Epidemiologist/statistician	26 [of 32] (81)		20 [of 25] (80)		6 [of 7] (86)	
Information specialist	19 [of 32] (59)		15 [of 25] (60)		4 [of 7] (57)	
Other	27 [of 32] (84)		22 [of 25] (88)		5 [of 7] (71)	
Responsible for/contributes to decision-making	15 (42)		12 (44)		3 (33)	
Technologies assessed/appraised						
Drugs (pharmaceuticals, biologicals, vaccines)	25 (69)		17 (63)		8 (89)	
Medical devices	36 (100)		27 (100)		9 (100)	
Diagnostics	31 [of 33] (94)		23 [of 25] (92)		8 [of 8] (100)	
Medical or surgical procedures	31 [of 33] (94)		23 [of 25] (92)		8 [of 8] (100)	
Other technologies	27 [of 35] (77)		20 (74)		6 [of 8] (78)	
Organisational or administrative systems	18 (51)		17 (63)		1 (13)	
Public health interventions	18 [of 35] (51)		12 (44)		6 [of 8] (75)	
Use of medical device classification system	18 (50)		15 (56)		3 (33)	
	*N*	Median (range)	*N*	Median (range)	*N*	Median (range)
Average duration of assessment work (months)						
Rapid review/response documents	14	1.0 (0.2–6.0)	11	(0.2–6.0)	3	1.2 (1.0–1.5)
Brief technical documents	7	2.2 (1.5–8.0)	5	(1.5–8.0)	2	2.9 (2.2–3.5)
Complete HTA or economic evaluation	18	9.0 (1.0–18.0)	14	10.5 (1.0–18.0)	4	8.2 (4.5–10.5)
Proportion of HTA reports assessing MDs	19	25% (5%–100%)	14	51% (5%–100%)	5	10% (5%–20%)

a All proportions based on the *N* = 36 (all agencies), *N* = 27 (MD specific agencies), *N* = 9 (Non MD specific agencies) unless otherwise indicated.

b A positive response could be given to more than one item.

### Process

A summary of agencies' processes for conducting HTA is presented in Table 2. As with structure, the majority of process characteristics appeared to be very similar across MD-specific and non–MD-specific agencies. However, there were some areas of difference. Compared with non–MD-specific agencies, MD-specific agencies were more likely to have a structured process (38 percent versus 80 percent), an internal unit (25 versus 67 percent) responsible for priority setting, to prioritize based on patient outcomes (33 percent versus 59 percent) and budget impact (33 percent versus 64 percent) and to externally commission reports (25 percent versus 44 percent).

**Table 2.** Summary of HTA Process Across Non-EU Agencies

			Non-MD
		MD Specific	Specific
	All Agencies	Agencies	Agencies
	(*N* = 36)	(*N* = 27)	(*N* = 9)
Characteristics	*N* (%)^a^	*N* (%)^a^	*N* (%)^a^
Process for priority setting	23 [of 33] (70)	22 (80)	3 (38)
The unit responsible for priority setting is:			
Internal Unit (e.g., Executive board, scientific committee)^b^	18 [of 32] (56)	16 [of 24] (67)	2 (25)
External Unit (e.g., government, insurance company)^b^	20 [of 32] (63)	15 [of 24] (54)	7 (88)
Technologies are selected/prioritised for assessment by:			
Perceived impact on patient outcomes^b^	15 [of 28] (54)	13 [of 22] (59)	2 [of 6] (33)
Budget impact of the technology^b^	16 [of 28] (57)	14 [of 22] (64)	2 [of 6] (33)
Prevalence of medical condition	16 [of 28] (57)	13 [of 22] (59)	3 [of 6] (50)
Assessment feasibility (e.g., available data, funding)^b^	9 [of 28] (32)	8 [of 22] (36)	1 [of 6] (17)
Any new technology^b^	1 [of 28] (4)	1 [of 22] (5)	0 [of 6] (0)
Selected new technology^b^	12 [of 28] (43)	10 [of 22] (45)	2 [of 6] (33)
Technology identified by external stakeholders^b^	19 [of 28] (68)	15 [of 22] (68)	4 [of 6] (67)
Other^b^	9 [of 28] (32)	7 [of 22] (32)	2 [of 6] (33)
The assessment work is performed by			
Mainly in-house HTA staff	25 [of 34] (74)	19 (72)	7 (78)
Mainly outsourced	7 [of 34] (21)	5 (19)	2 (22)
Other	2 [of 34] (6)	3 (11)	0 (0)
The organisation			
Commissions reports^b^	13 [of 33] (39)	11 [of 25] (44)	2 [of 8] (25)
Commissioned to perform reports^b^	25 [of 33] (76)	18 [of 25] (72)	7 [of 8] (88)
The assessments are based on			
Submissions from external stakeholders^b^	24 [of 32] (75)	18 [of 25] (72)	6 [of 7] (86)
Internally conducted research^b^	14 [of 32] (44)	11 [of 25] (44)	3 [of 7] (43)
Products or services produced by the organisation:			
Reports for decision makers^b^	32 [of 34] (94)	23 [of 25] (92)	9 (100)
Research projects with primary data^b^	11 [of 34] (35)	9 [of 25] (36)	3 (33)
Academic and training activities^b^	19 [of 34] (56)	12 [of 25] (52)	6 (67)
Clinical practice guidelines^b^	13 [of 34] (38)	10 [of 25] (40)	3 (33)
Other^b^	7 [of 34] (21)	6 [of 25] (24)	1 (11)
Types of HTA reports produced			
Full HTA report^b^	24 [of 30] (83)	20 [of 24] (83)	5 [of 6] (83)
Rapid HTA report^b^	20 [of 30] (67)	15 [of 24] (63)	5 [of 6] (83)
Other^b^	19 [of 30] (63)	14 [of 24] (58)	5 [of 6] (83)
Assessment results are made publically available on			
Agency web site^b^	26 [of 35] (74)	20 [of 27] (74)	6 [of 8] (75)
INAHTA database^b^	12 [of 35] (34)	10 [of 27] (37)	2 [of 8] (25)
Other^b^	14 [of 35] (40)	11 [of 27] (44)	2 [of 8] (25)
Stakeholders (i.e., clinical specialist, patients’ associations, industry) role in the HTA process:			
Trigger HTA process^b^	21 [of 34] (62)	17 [of 26] (65)	4 [of 8] (50)
Involved in the assessment process^b^	16 [of 34] (47)	11 [of 26] (42)	5 [of 8] (63)
Involved in the appraisal process^b^	12 [of 34] (35)	10 [of 26] (38)	1 [of 8] (25)
Involved in reimbursement/pricing decisions^b^	7 [of 34] (21)	6 [of 26] (23)	1 [of 8] (13)
External review of HTA assessments	27 [of 33] (82)	21 [of 21] (81)	6 [of 7] (86)
Have specific measures to ensure transparency of HTA process (e.g. declaration of conflict of interest)	21 [of 23] (91)	18 [of 19] (95)	3 [of 4] (75)
Repeat assessments of given technologies in regular intervals	15 [of 29] (52)	14 [of 23] (61)	1 [of 6] 17
The individual or body responsible for technology funding/coverage/policy decision at end of HTA process			
A committee within the same organization	7 [of 33] (21)	6 [of 25] (24)	1 [of 8] (12)
A different organisation	26 [of 33] (78)	19 [of 25] (76)	7 [of 8] (88)
HTA is mandatory for decision making	10 [of 33] (31)	11 [of 25] (44)	0 [of 8] (0)
Decision-making body can deviate from HTA results	30 [of 31] (97)	22 [of 23] (96)	8 [of 8] (100)

a All proportions based on the *N* = 36 (all agencies), *N* = 27 (MD specific agencies), *N* = 9 (Non MD specific agencies) unless otherwise indicated.

b A positive response could be given to more than one item.

### Methods

Table 3 summarizes the scientific methods adopted by HTA agencies. Sixty nine percent of all agencies had a methods guidance document or policy for evidence submission. However, only one agency, the Department of Science and Technology in Brazil, reported that they have developed scientific methods guidelines specifically for the HTA of MDs. No agencies stated that they were in process of developing MD-specific methods guidance. Areas of difference between MD-specific and non–MD-specific agencies were: consideration of organization aspects (63 percent versus 33 percent), observational studies (35 percent versus 14 percent), expert opinion (52 percent versus 14 percent), and MD-specific attributes (52 percent versus 33 percent), the use of cost consequence (48 percent versus 14 percent) and cost minimization methods of economic evaluation and specific processes to ensure transferability (47 percent versus 0 percent).

**Table 3.** Summary of HTA Methods of Non-EU Agencies

	All Agencies	MD Specific	Non-MD Specific
	(*N* = 36)	Agencies (*N* = 27)	Agencies (*N* = 9)
Characteristics	*N* (%)^a^	*N* (%)^a^	*N* (%)^a^
Guidance document/policy for methods of evidence submission and/or methods of conduct for assessment	22 [of 32] (69)	17 [of 24] (71)	5 [of 8] (63)
Methods guidance available on website	15 [of 30] (50)	12 [of 22] (55)	3 [of 8] (38)
Comparators used during assessment			
Placebo^b^	6 [of 29] (21)	5 [of 24] (21)	1 [of 5] (20)
All relevant health technologies^b^	29 [of 29] (100)	24 [of 24] (100)	5 [of 5] (100)
Evolution stage at which technologies are assessed			
Emerging/new technology^b^	33 [of 34] (97)	25 [of 25](100)	8 (89)
Established or widespread practice^b^	25 [of 34] (74)	17 [of 25] (68)	8 (89)
Declining use in practice^b^	8 [of 34] (24)	8 [of 25] (32)	0 (0)
Elements of technology evaluated			
Health problem and current use of technology	29 [of 36] (81)	22 [of 27] (81)	7 (78)
Description/technical characteristics of technology	29 [of 36] (81)	21 [of 27] (78)	8 (89)
Safety	35 [of 36] (97)	27 [of 27] (100)	8 (89)
Clinical effectiveness	35 [of 36] (97)	26 [of 27] (96)	9 (100)
Costs and economic evaluation	34 [of 36] (94)	26 [of 27] (96)	8 (89)
Ethical aspects	23 [of 36] (64)	18 [of 27] (67)	5 (55)
Organisational aspects	20 [of 36] (56)	17 [of 27] (63)	3 (33)
Legal aspects	10 [of 36] (28)	7 [of 27] (26)	3 (33)
Social aspects	17 [of 36] (47)	11 [of 27] (41)	6 (67)
Assessment methodologies adopted			
Systematic review	31 [of 33] (94)	24 [of 25] (96)	7 [of 8] (88)
Meta-analysis	24 [of 33] (73)	19 [of 25] (76)	5 [of 8] (63)
Clinical evaluation	18 [of 33] (55)	15 [of 25] (60)	3 [of 8] (38)
Trials (observational)	9 [of 30] (30)	8 [of 23] (35)	1 [of 7] (14)
Trials (interventional)	9 [of 30] (30)	7 [of 23] (30)	2 [of 7] (29)
Expert opinion	13 [of 30] (30)	12 [of 23] (52)	1 [of 7] (14)
Economic analyses	27 (82)	21 (84)	6 (75)
Clinical trial based economic evaluation	14 (43)	11 [of 23] (48)	2 [of 7] (29)
Decision model based economic evaluation	27 (83)	20 [of 23] (87)	5 [of 7] (71)
Consideration of MDs specific attributes	17 (47)	14 (52)	3 (33)
Type of analyses allowed/recommended in full economic evaluation			
Cost-effectiveness	27 [of 28] (96)	20 [of 21] (95)	7 [of 7] (100)
Cost-utility	22 [of 28] (79)	17 [of 21] (81)	5 [of 7] (71)
Cost consequences	11 [of 28] (39)	10 [of 21] (48)	1 [of 7] (14)
Cost minimization	12 [of 28] (43)	10 [of 21] (48)	2 [of 7] (29)
Other	4 [of 28] (14)	4 [of 21] (19)	0 [of 7] (0)
Perspectives considered for economic analyses			
Societal	12 [of 29] (41)	10 [of 23] (43)	2 [of 6] (33)
Third party payer	17 [of 29] (59)	13 [of 23] (57)	4 [of 6] (67)
Other	7 [of 29] (24)	6 [of 23] (26)	1 [of 6] (17)
Estimation of uncertainty (e.g. confidence intervals) considered	11 [of 23] (48)	7 [of 17] (41)	4 [of 6] (67)
Public thresholds used to determine cost-effectiveness	4 [of 28] (14)	2 [of 22] (9)	2 [of 6] (3)
National specific data mandatory	8 [of 27] (30)	6 [of 22] (29)	1 [of 6] (33)
Formally uses HTA evaluations conducted by other organisations or countries	18 [of 28] (64)	12 [of 21] (57)	6 [of 7] (86)
Specific processes to ensure transferability	7 [of 17] (41)	7 [of 15] (47)	0 [of 2] (0)

a All proportions based on the *N* = 36 (all agencies), *N* = 27 (MD-specific agencies), *N* = 9 (non–MD-specific agencies) unless otherwise indicated.

b A positive response could be given to more than one item.

#### Stage 2: Semi-structured Interviews

All twenty-seven HTA agencies that were judged in stage 1 to be MD-specific were invited to participate in a follow up interview process (stage 2). Eighteen (67 percent) agencies agreed to be interviewed. In most cases, the interviews were conducted by telephone with the same agency individual(s) who verified the Web assessment questionnaire. In one case, a telephone interview was not possible and instead responses were obtained by e-mail. After an iterative process of qualitative data analysis, the following seven broad themes were identified: (i) capacity; (ii) complex intervention and co-dependency; (iii) decision making; (iv) evidence; (v) coordination; (vi) decision problem; (vii) transferability. In a next step, these themes were subsumed under the three dimensions of HTA used earlier in this report (i.e., structure, process and methods). A tabulated summary of the results of the semi-structured interviews is available in Supplementary Table 1.

### Structure

#### Decision Making

Some interviewees indicated that their organizational structure effectively evolves to meet the needs of decision makers as it relates to HTA of MDs. By adapting the organizational structure according to the objectives outlined in policy requirements rather than by an HTA “codebook,” agencies contend that they are better positioned to efficiently inform key funders about the medical, social, economic, and ethical issues associated with introducing a new technology into the health care system. One interviewee from an MD-specific agency purported:

*“The structure of the HTA has evolved to meet the funding programs; it directly reflects that need and not the other way round (not build on an HTA-centric view). HTA serves the need of the policy requirement and its organizational structure fits with what is expected from it.”*

They find that this approach reduces the time needed to translate MD-specific evidence into the health system.

#### Capacity

Many interviewees cited insufficient resources (e.g., budget, skilled economists) as a limitation, creating hurdles in the agency's ability to adapt to policy needs for HTA of MDs. For example, one key informant explained that the lack of resources has a trickle-down effect on the way in which their agency is able to examine MD evidence:

*“Not enough [MD] experts – there is a very large gap there. So we only look at evidence in the sense of systematic reviews, meta-analysis.”*

### Process

#### Capacity

Some interviewees indicated that they integrate external academic partners into the HTA process for MDs when they do not have staff with the appropriate skills to perform evidence assessments. In fact, the use of external networks was even cited as an asset by some interviewees. For example:

*“I think some of the real operational issues related to medical technologies are important to understand – How this piece of equipment works in the hospital, who's using it, what happens if it's in a different pair of hands? I think we don't necessarily have a lot of that expertise in house. I'd say one of our strengths is building those networks.”*

#### Coordination

Some interviewees noted the value of integrating subject matter experts in the HTA process for MDs. The input of experts fosters stakeholder buy-in on policy decisions resulting from evidence appraisals. Furthermore, it supports the contextualization of MD evidence and the introduction of MDs into the health system.

Another issue identified by many interviewees is that the introduction of MDs into the healthcare system is diverse and not standardized:

*“For devices, our health care system is extremely fractured. Devices can come into our system at a regional level, hospital level, within a particular doctors' office, at a provincial level, or even at a national level. So, for devices we have an extremely fractured system for entry points. Because of that, we have different kinds of evidence requirement at different kinds of levels. I would go so far to say at some levels there is no rigorous evidence assessment taking place.”*

Although many interviewees acknowledge that the procedure used to review devices, including evidence requirements, is inextricably linked to the point of entry into the health care system, they expressed discontent with the poor levels of evidence required for pre-market access for MDs.

Many also found fault with the level of harmonization between the regulatory and reimbursement bodies' evidence requirements. In consequence, this imbalance leads to unnecessarily prolonged review processes.

*“The fact of having an evaluation of sanitary registration for marketing that does not use the same principles as are used for incorporation in the Public Health System, are considerable obstacles in HTA for MD.”*

#### Complex Interventions/co-dependency

Another topic was how agencies manage complex interventions, that by definition include MDs, and the evidence assessment process for co-dependent technologies, for example, delivery of drug using a device technology. Responses were suggestive in nature, including recommendations on how to improve the management of MDs in complex environments as well as harmonize the review process by having co-dependent technologies simultaneously reviewed by drug and device assessment committees.

#### Decision Making

Some agencies noted that the approval channels integrated into their standard operating procedures mitigate potential challenges associated with translating MD evidence into policy. For example, one key informant describes current review processes as sufficient for advising decision makers on whether to adopt a new technology:

*“In health technology assessment term, I think there is not any raised problem of HTA of MDs. The applied HTA of MDs has been discussed and assessed by sub-committee which consist of qualified medical doctors in their specialty, and has been re-discussed internally whether to accept, modify or reject the assessment of sub-committee.”*

### Methods

#### Evidence

Most interviewees stated that there is a lack of methodological guidance for MDs. Furthermore, they identified several shortcomings with evidence requirements for MDs.

*“The main problem of health technology assessment is systematic literature review that search, analyze and contemplate existing studies […] The main problems of literature review are that any newly emerging technologies which have potential benefit for patients but lack of literature evidence will not be approved as new technologies.”*

The quality of evidence (e.g., lack of randomized trial evidence) was cited several times as the main challenge for performing HTA of MDs.

*“The main challenge is that there is not enough good evidence […] I think that there is a broad societal question of how we will ever collect enough data to answer all the questions we need to answer. I think that that's really the biggest challenge.”*

The quality of evidence was also described as being a limiting factor for the scope of device assessments.

*“There are certainly again limitations on what we can do with the devices given the nature of the evidence base.”*

The MD industry was identified by some interviewees as a “conspirator” to poor evidence generation. As a result, agencies experienced challenges with obtaining the appropriate evidence for HTA of MD.

#### Capacity

Some interviewees identified human resource constraints as a great challenge when attempting to apply scientific methods to critique MD evidence. The range of staff skills tend to be limited. An interviewee described how they are addressing skill deficiencies such as the ability to appropriately evaluate various types of evidence or data.

*“We've also recognized the need to put a real focus in terms of getting our staff comfortable with qualitative research. I'm not sure we'll get it to where it needs to be in the next year, but at least getting people to understand what qualitative research can bring to an assessment on the non-drug side and especially with respect to the non-clinical, non-economic of HTA. Within the team that we have here, we're working to build awareness of what it is and why it's important and then to be able to take on some projects to get people actually using it and doing it and then get their comfort level up.”*

#### Transferability

Many interviewees identified the inability to generalize the findings obtained from MD evidence reviews to their local context as highly problematic.

*“The methodology of HTA of MDs can be really difficult. All we know is generally translated from drugs to devices but we know that differences exist, for instance the performance of devices in clinical practice can be very different from that assessed in controlled setting.”*

This weakness was attributed to device-specific challenges, such as the learning curve. Interviewees believed that intervention evidence, typically generated in environments with highly skilled staff, may not be representative of the health system where the technology will be introduced:

*“Such another issue, an issue that probably exists here and elsewhere is that the early use and early evidence on devices tend to be in individuals with “the best hands problem”- so specialized academic centers that either develop technologies themselves or have used it for a number of years. So it's very difficult to then generalize what the findings might be in the community.”*

## DISCUSSION

### Main Findings of the Study

In addition to providing a detailed survey of the practices and methods of a comprehensive sample of non-EU HTA agencies, this study specifically sought to characterize and compare current HTA activities for MDs across these agencies. In particular, we assessed agencies in terms their organizational structure, processes, and scientific methods. All 36 HTA agencies identified within our sampling framework undertook the evaluation of MDs. A substantive proportion (27/36; 75 percent) of HTA agencies reported that they had developed specific approaches within their organizational structure or procedures for the assessment or appraisal of MD technologies (“MD-specific agencies”). For example, organizational developments included the allocation of specific staff to MDs assessment or the setup of a completely separate program or unit within the agency for device evaluation. Procedural developments could include convening a specific committee to appraise MDs evidence and provide policy advice. There appeared to be few differences in the organization, process, and methods of between MD-specific and non–MD-specific agencies. Although several agencies had a methods guidance document or policy for evidence submission, only one MD-specific agency (Department of Science and Technology in Brazil) had developed specific scientific methodological guidance for the HTA of MDs (20).

Our interviews confirmed several commonly cited challenges in the HTA of MDs (9–11). These included: the relatively poor quality of evidence for MDs, problems in generalizing the MD evidence obtained in a specific setting to another, the “learning curve” effect and difficulties in defining the scope of the appropriate decision problem when conducting HTAs of MDs. This later challenge is particularly emphasized by the recent national review of HTA in Australia in 2011 (21). This review has led to reform the processes by which the MSAC undertake MDs evaluation, including the introduction of a formal scoping workshop that brings together key stakeholders (i.e., industry, clinicians, and patients) to have the opportunity to debate and finalize the scope of the decision problem (e.g., in terms of the population, comparator(s), and key outcomes) before the state of the actual assessment of evidence.

That the vast majority of agencies identified in this survey has not developed MD-specific methods guidelines indicates that these agencies applied the same methodological approach to the assessment of MDs evidence as non-device technologies, such as drugs. However, we did identify an innovative approach to evidence generation for MDs. The MaRS Excellence in Clinical Technology Evaluation (EXCITE) program recently established in Ontario (22), has been developed to integrate research and development directly into the health care system to collect the evidence necessary to support “parallel submission” with both regulators and HTA decision-making authorities. At the time we completed our survey, the EXCITE program was recruiting patients for its first randomized controlled trial of a MD (Symplicity^TM^ catheter renal ablation therapy for treatment-resistant hypertension), specifically designed to facilitate parallel submission to Health Canada, for regulatory purposes, and to the Ontario Health Technology Advisory Committee (23). Other ongoing EXCITE projects include electrical stimulation for upper limb movements in stroke patients, home sleep apnea events detector, and RNA disruption assay for early prediction of complete response to chemotherapy in breast cancer patients (24).

### Comparison to Previous Literature

Whereas there have been several previous surveys of international HTA agency practice, we believe this to be the first international survey of HTA practice to focus on MDs evaluation. Three previous studies have included aspects of their review of HTA practice to the consideration of MDs. In 2012, Stephens et al. published a survey of thirty HTA organizations representing sixteen countries including Europe, North and South America, and Australia (5). In accord with our findings, this report found that the majority of respondents assessed MDs: 94 percent in Europe and 76 percent in the United States. Similarly, thirty of the forty-one (75 percent) HTA organizations responding to a EUNetHTA survey reported to assess MDs (6). In contrast, the 2009 survey by Charles River Associates reported that almost half of the HTA systems (7/15; 47 percent) did not produce any HTA reports for MD technologies, unlike reports considering drugs that were published by all HTA systems (8). The lower level of MD HTA activity identified in this later survey can be explained by the sample of HTA agencies which mainly consisted of drug specific HTA agencies (e.g., Pharmaceutical Benefits Advisory Committee (PBAC) in Australia, Pharmaceutical Management Agency (PHARMAC) in New Zealand, The Dental and Pharmaceutical Benefits Agency (TLV) in Sweden). In addition, other components of HTA activity within those same countries were ignored (i.e., MSAC and Australian Safety and Efficacy Register of New Interventional Procedures-Surgical (ASERNIP) in Australia, New Zealand Health Technology Assessment (NZHTA) in New Zealand, Swedish Council on Technology Assessment in Health Care (SBU) in Sweden).

### Strengths and Limitations

The main strengths of this study include the comprehensive and systematic approach to the selection of HTA agencies, high levels of response both to the request for verification of our agency Web-site surveys and interview invitations, and quality assurance measures for data collection (development of structured questionnaire to assess agency Web sites and a second researcher to verify the all Web-site based assessments and interviews).

However, we acknowledge several potential limitations. As the scope of this survey was limited by the funder of the project to consideration of non-EU HTA agencies, our findings may not be directly generalizable to the EU jurisdiction. A consequence of the comprehensiveness of our survey was the inclusion of a heterogeneous group of HTA agencies (e.g., size, age). Although we largely took a summative (“average”) approach to the presentation of results across agencies, where possible, we did report ranges. The assessment of agency Web sites for some countries (e.g., Norway) was limited to text and material available in English, excluding content in other languages. However, to minimize the risk of this retrieval bias, key informants reviewed the accuracy of the country-level analysis and Web-based assessment form. An alternative approach that may have led to more direct collection of data would have been to directly request agencies to self-complete a questionnaire. Although this later approach has been undertaken by several international surveys of HTA agencies, it has the major limitation of a low response rate. For example, the ISPOR and EUNetHTA surveys that both used questionnaires reported response rates of only 42 percent and 36 percent, respectively (5;6). Finally, to compare the approaches of HTA of MDs with drugs, a contrast between so-called “MD-specific” agencies versus “non–MD-specific” HTA agencies (i.e., *between* agency comparison) was conducted. It is possible that a *within* agency analysis (i.e., comparison of structure, processes, and methods of the MD and non-MD technology assessment within the same agency) may have been more sensitive to detecting differences. However, the data collection methods were not designed to undertake such a within agency comparison of HTA approaches as the available Web-site material does not often differentiate between the evaluation of differing health technologies.

### Implications for Policy

Our findings raise some important implications. First, in contrast to the regulatory requirements that appear fairly consistent across international settings (25), we found that HTA organizational structures, processes, and scientific methods for MDs can vary considerably across countries. Second, although it is well accepted that MDs differ from other health technologies, little evidence of differentiation in the HTA methods used by agencies for devices compared with drugs was found. This raises the question of whether these differences are such to require a fundamentally different methodological approach to the HTA of MDs compared with drugs or, instead, specific organizational or procedural adaption to current HTA activities, to consider MD-specific issues. Scientific methods to deal with usual challenges posed by MDs evaluation are being developed (26–28) and are part of the armamentarium of HTA analysts regardless of the type of technologies evaluated. Third, for other jurisdictions to develop innovative MD evaluation initiatives, such as Ontario's EXCITE programs, several factors need to be in place. For example, an infrastructure must exist to support trial development. The EXCITE program was introduced in a setting where academic centers with experience in field evaluation and coverage-with-evidence development activities already existed. Another important requirement is that a mutual understanding must exist between the various stakeholders, and in particular, the different incentives between device manufacturers, academics, and policy makers must be aligned. Finally, appropriate financing is necessary to support such activities. As many interviewees have noted, the issue is more about capacity building and training on advanced methodological tools for HTA MD assessors. The on-going EXCITE MD trial is funded by a device manufacturer although, importantly, the data collection, analysis and reporting of the studies is overseen by an independent Trial Steering Committee (23).

### Conclusions

This survey provides a unique overview of organization and practices of non-EU HTA agencies. It is widely cited that MDs differ from other health technologies, in particular, they can change rapidly, clinical outcomes often depend on training and experience of operator, and pricing is more dynamic than drugs. The fact that all the non-EU HTA agencies included in this survey undertake the evaluation of MDs and do so alongside to other health technologies such as pharmaceuticals, supports the principles published by Drummond and colleagues for good HTA practice: “HTA should include all relevant technologies” (14). However, given the lack of evidence for differentiation in scientific methods of HTA used by agencies for evaluation of devices compared with drugs, our study raises the question of whether the differences between medical technologies are such to require a fundamentally different methodological approach to their HTA, and whether organizational or procedural adaptation may suffice to accommodate specific challenges posed by devices' evaluation. In other words, existing primary and secondary scientific methods, including the analysis of observational studies and evaluation approaches for complex interventions (26–28), may provide a sufficient methodological basis for the HTA of MDs.

## Supplementary material

For supplementary material accompanying this paper visit http://dx.doi.org/10.1017/S0266462315000185. click here to view supplementary material

## CONFLICTS OF INTEREST

All authors declare no conflict of interest.
