# Supplementary material for: HEALTH TECHNOLOGY ASSESSMENT OF MEDICAL DEVICES: A SURVEY OF NON-EUROPEAN UNION AGENCIES
Source: Int J Technol Assess Health Care. 2015;31(3):154–65. doi: 10.1017/S0266462315000185 (PMC4535322; doi:10.1017/S0266462315000185)
Supplement: Supplementary file 1 [file S0266462315000185sup001.docx]

eFigure 1 Summary of two stage survey process


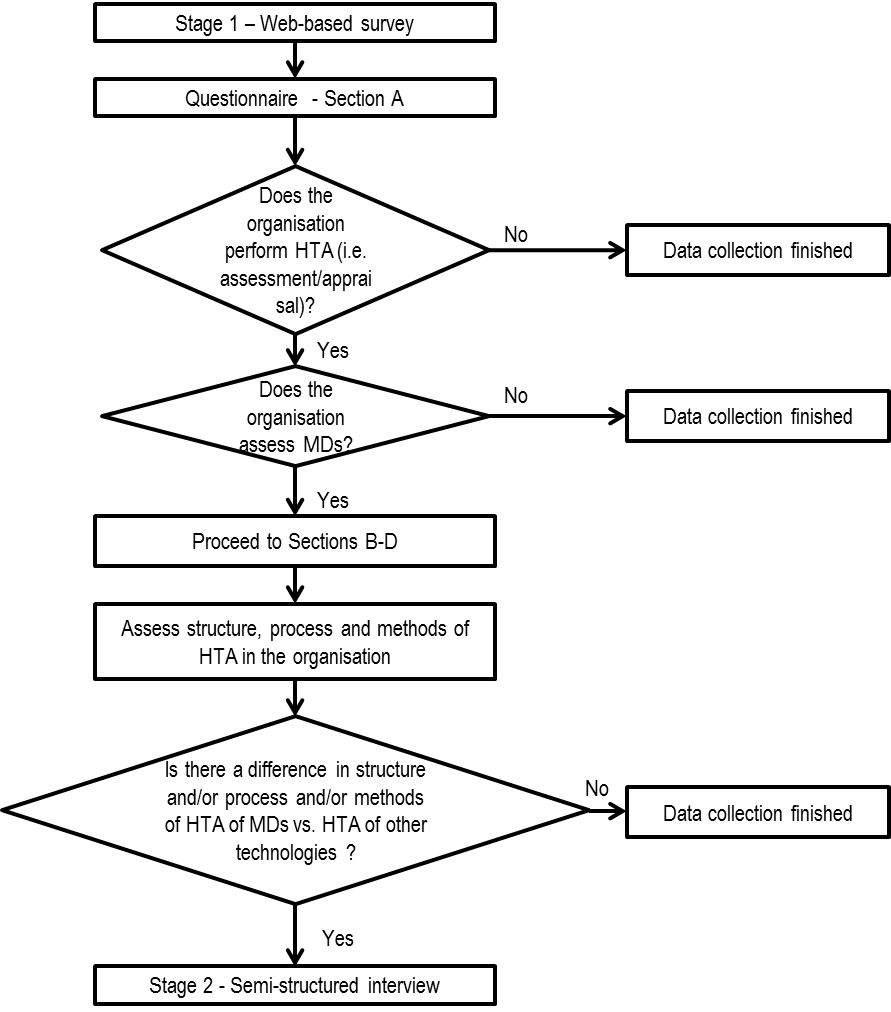


eFigure 2. Selection Sampling of non-EU HTA organisations


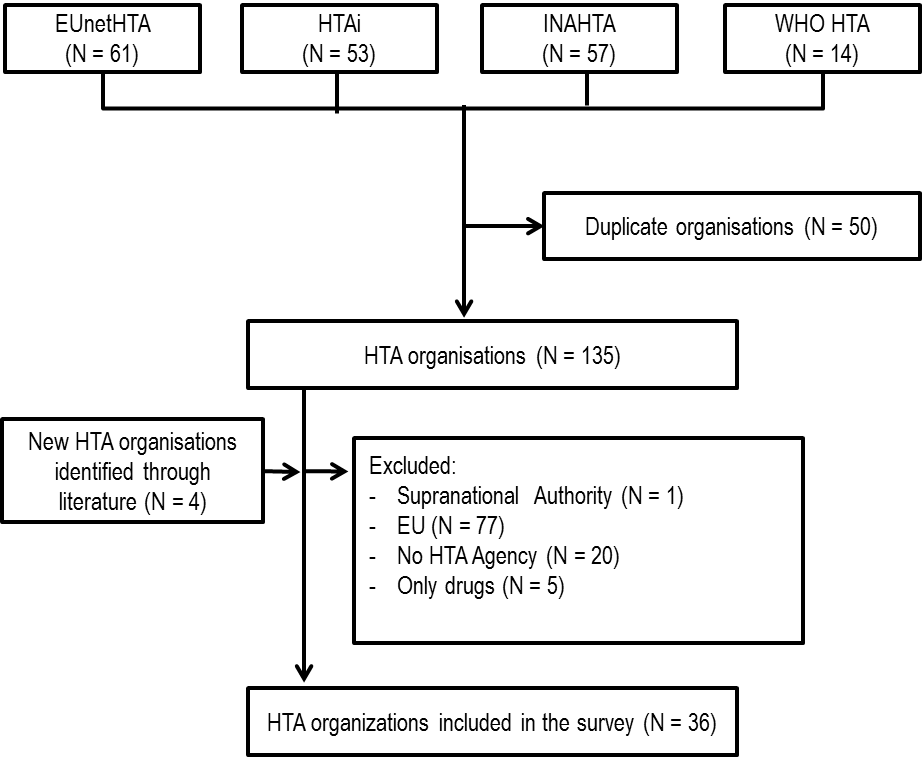


EUnetHTA = European network for health technology assessment; HTAi = Health Technology Assessment International; INAHTA = International Network of Agencies for Health Technology Assessment; WHO HTA = World Health Organization HTA Collaborating Centres.

eTable 1. Qualitative interview data analysis: summary of key points addressing the issues associated with HTA of medical devices

| **HTA perspectives** | **Value** | **Key Point Summary** | **Categorisation^a^** |
| --- | --- | --- | --- |
| **Structure** | ***Positive*** | ***Decision Making***   - The framework in place has evolved to meet the needs of funding programmes. | Some |
|  | ***Negative*** | ***Capacity***   - Insufficient resources (e.g., budget, economists, and pharmacists) create challenges for organisation to deliver according to its purpose. | Many |
| **Process** | ***Positive*** | ***Capacity***   - Experts from external networks augment agencies’ capability to meticulously review applications. | Some |
|  |  | ***Coordination***   - Integrating feedback from external parties (e.g., patients, clinicians) facilitates stakeholder buy-in and informs the contextualization of data. | Some |
|  |  | ***Decision Making***   - Standard operating procedures (i.e., approval channels via subcommittees) support the translation of evidence into policy. | Some |
|  | ***Negative*** | ***Coordination***   - Heterogeneity of devices makes the coordination of evidence assessment on different system levels difficult. - Disconnect between the regulator and reimbursement body adversely impacts the timeframe of HTAs. | Many |
| **Methods** | ***Positive*** | ***Evidence***   - Consistent application facilitates sound evidence synthesis and appropriately informs appraisal. | Some |
|  | ***Negative*** | ***Capacity***   - Staff lacks skills to appropriately assess various types of data (e.g., RCT, qualitative). | Some |
|  |  | ***Transferability***   - Cannot generalise findings due to variation of (local) social context. | Many |
|  |  | ***Evidence***   - Poor quality evidence limits the ability to answer desired questions. - Industry is unclear about the definition of [adequate] evidence. | Most |

1. ^“Some” corresponds to key points made by 10-30% of interviewees, “Many” corresponds to key points made by 31-65% of interviewees, and “Most” corresponds to key points made by 66-100% of interviewees^
